# The Effect of Hemostatic Agents on the Retention Strength of Zirconia Crowns Luted to Dentin Abutments

**DOI:** 10.3390/ma12060979

**Published:** 2019-03-25

**Authors:** Christian Maischberger, Anja Liebermann, Bogna Stawarczyk

**Affiliations:** Department of Prosthetic Dentistry, University Hospital, LMU Munich, Goethestrasse 70, 80336 Munich, Germany; Christian.Maischberger@gmx.de (C.M.); Bogna.Stawarczyk@med.uni-muenchen.de (B.S.)

**Keywords:** hemostatic agent, astringent, retention strength, zirconia, adhesive luting

## Abstract

The purpose of this study was to investigate whether hemostatic agents (HA) show an effect on the retention strength (RS) of zirconia crowns luted to dentin abutments after cleaning with an air/water spray. Human molars (N = 60/n = 12) were prepared and zirconia crowns were milled. Prior to luting (Scotchbond Universal/RelyX Ultimate), molars were pretreated using HA: i. 25% AlCl_3_ (VSC), ii. 20% Fe_2_(SO_4_)_3_ (VS), iii. 15.5% Fe_2_(SO_4_)_3_ (AS), iv. 12.7% iron ion solution (ASX) and v. no pretreatment (control). Mastication simulation and pull-out tests were performed. Failure types were defined: cohesive 1—tooth root; cohesive 2—tooth crown; adhesive 1—cement on tooth; adhesive 2—cement on tooth and crown; mixed—adhesive/cohesive. Data were analyzed using 1-way ANOVA, post-hoc Scheffé, Pearson’s chi-square-test and Ciba–Geigy table (p = 0.05). No RS differences between the tested groups were observed (p = 0.200). ASX fractured more cohesive 2 than the control group. VSC showed more cohesive 2 than adhesive 1 fractures. VS showed more adhesive 2 than mixed fractures. AS showed more cohesive 2 than adhesive 1 and more adhesive 2 than mixed fractures. ASX showed predominantly cohesive 2 fractures. RS was not affected when HA were cleaned off by 30 s of air/water spray prior to luting. HA still seem to weaken the dentin abutment, making it prone to cohesive fractures.

## 1. Introduction

The adhesive bond strength to dentin depends on the formation of an appropriate hybrid layer by the incorporation of resin tags into the dentinal tubules [1,2]. To achieve a high-quality bonding, the creation of a contaminant-free working field is of highest importance. Contaminants like blood or saliva may occlude dentinal tubules and therefore have a detrimental effect on hybrid layer formation with a decrease in bond strength for adhesive procedures [3,4,5,6].

Dentists often have to deal with blood and sulcular fluid flow as a result of trauma to the gingival tissues as occurring by para- or sub-gingivally placed preparation margins. In such situations, gingival tissue management in terms of hemorrhage control is important [2]. Therefore, hemostatic agents (HA), containing different active ingredients like aluminum chloride (AlCl_3_) or ferric sulfate (Fe_2_(SO_4_)_3_), are often used to achieve hemostasis. During their application these agents also come in contact with hard dental tissues, on which they may exert possible negative effects. Several studies demonstrated the partial or complete removal of the dentinal smear layer after application of HA caused by their high acidity [7,8,9,10].

Further studies showed residual particles of HA in form of crystalline growths or granular deposits left on the dentinal surface or even changes in hydroxyapatite crystals which seems to make them more resistant to acids [11,12]. Changes like these in the dentinal surface or smear layer may reduce the dentin’s susceptibility to acid etching or may obliterate dentinal tubules. Both changes affect the formation of the hybrid layer, eventually reducing the bond strength between adhesive and dentin [11,12,13,14]. Nevertheless, to date there is no general consensus if HA adversely affect adhesive systems. Several studies were conducted investigating the influence of HA on self-etch and total-etch adhesive systems, showing controversial results. Most studies concerning self-etch adhesives (SEA) demonstrated a decrease in bond strength to dentin after contamination with HA [9,12,14,15,16,17,18]. However, some authors reported no decrease in bond strength between SEA and dentin [10,16,19]. In contrast to SEA, studies concerning the effect of HA on the bond strength of total-etch adhesive systems to dentin demonstrated more consistent results of no decrease of bond strength after phosphoric acid etching [9,10,16,20]. In addition, two studies reported a decrease in bond strength with total-etch adhesives [15,21].

Due to the controversial outcomes of these previous studies it is not yet possible to draw definite conclusions. The reason for such contrasting results might be attributed to the lack of standardization of methodology used. Studies showed great differences in testing procedures, handling and duration of HA application, combination and amount of used materials, additional contamination with blood or saliva, and also in the dentin condition regarding storing protocols and types of dentin (human primary/human permanent/bovine teeth). Other studies in turn evaluated the influence of HA on the microleakage of class V composite resin restorations. In all studies, microleakage was increased when SEA were used [22,23,24,25]. In case of total-etch adhesives, no increase in microleakage was demonstrated [22,23].

Due to the fact, that studies still did not clarify the influence of HA on bond strength and often can’t be compared with real clinical situations, this study should investigate the influence of different HA on the retention strength (RS) of zirconia crowns luted to dentin abutments. The null hypothesis stated, that the RS between zirconia crowns and dentin abutment bonded by adhesive luting procedure is not affected by any of the HA tested.

## 2. Materials and Methods

**Figure 1:**
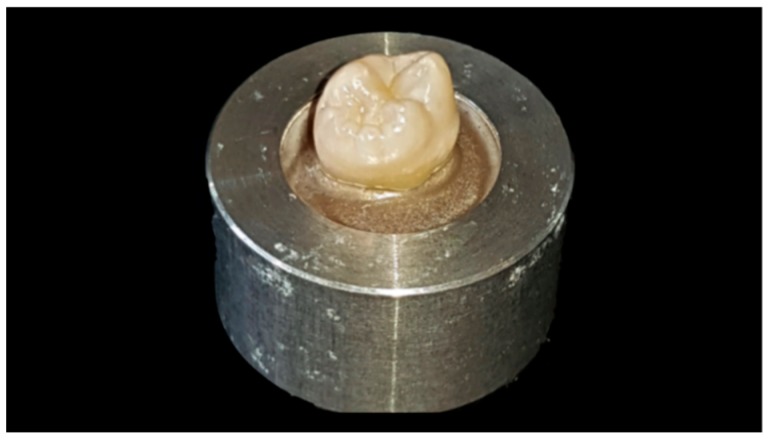
Tooth embedded into aluminum body, before preparation.

Human, freshly extracted, sound molar teeth (N = 60) were collected, disinfected for a maximum of 7 days in 0.5% Chloramine-T solution (Sigmar-Aldrich Laborchemikalien, Munich, Germany, CAS: 149358-73-6) and stored in distilled water at 5 °C for a maximum of 6 months. All teeth originated from an anonymous collection of several dentists located around Munich. As datasets cannot be related to human individuals, that means that the natural teeth used in this investigation cannot be traced to the patients anymore, no ethical–legal objections against the project were identified by the ethical committee according to the declaration of no objection of 10.07.2012. Teeth were cleaned and embedded (ScandiQuick, SCAN-DIA, Hagen, Germany) up to their cemento-enamel junction in round aluminum bodies (Figure 1).

**Figure 2:**
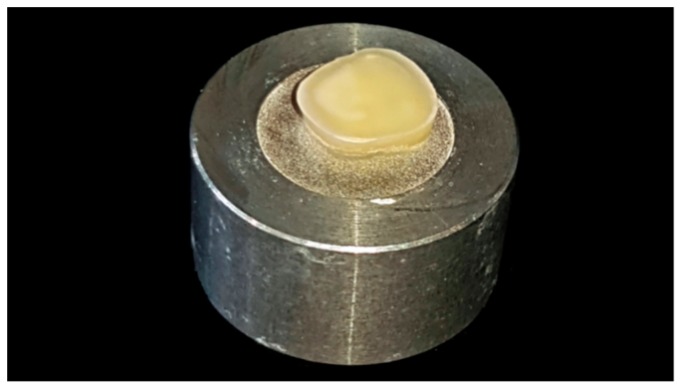
Occlusal part of tooth cut off, resulting in a horizontally parallel plane.

The tooth crown preparation was divided into two parts. First, a cutting machine with water-cooling (Secotom-50, Struers, Ballerup, Denmark) was used to cut off the occlusal part of the tooth forming a horizontally parallel occlusal plane (Figure 2).

**Figure 3:**
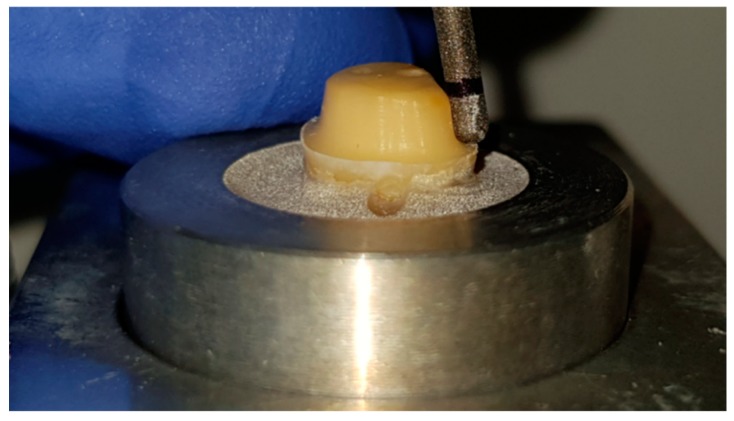
Tooth preparation in parallelometer.

Second, a parallelometer (F4 basic, Dentsply, Hanau, Germany) and a water-cooled turbine (Perfecta 900, W&H, Laufen, Germany) with a round-end straight diamond bur (GEBR. BRASSELER, Lemgo, Germany, Lot.No. 975066) were used to prepare the circular surface of the tooth to ensure a uniform preparation angle of 10° with a 1.0 mm cervical finish line (Figure 3).

**Figure 4:**
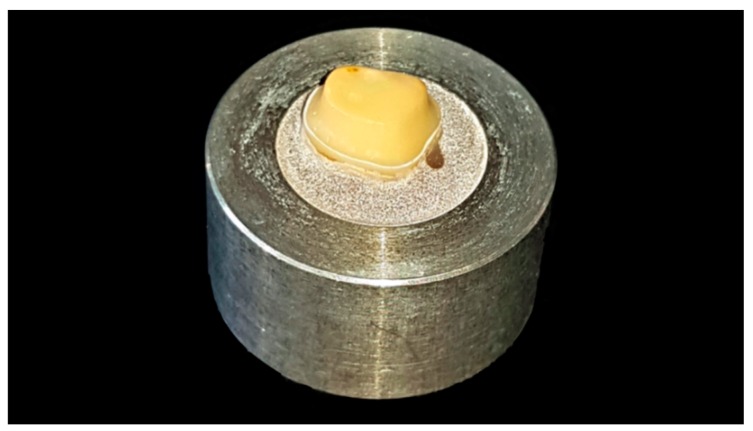
Finalized tooth preparation.

The burs were marked at 3 mm distance from the tip to ensure a standardized abutment height of 3 mm, finishing disks (Sof-Lex 1982C/ 1982M, 3M, Seefeld, Germany) were used to smoothen sharp edges (Figure 4).

**Figure 5:**
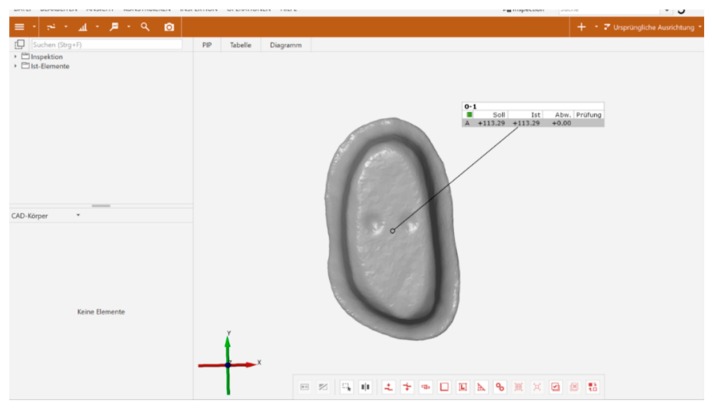
Surface calculation in mm² using GOM 3D analysis software.

The prepared teeth were scanned with a light scanner (Ceramill map400, Amann Girrbach, Koblach, Austria) and a STL (Standard Triangulation Language) file was created (Ceramill Mind, Amann Girrbach, Koblach, Austria) and the bonding surfaces were calculated (GOM Inspect, GOM, Braunschweig, Germany) (Figure 5).

**Figure 6:**
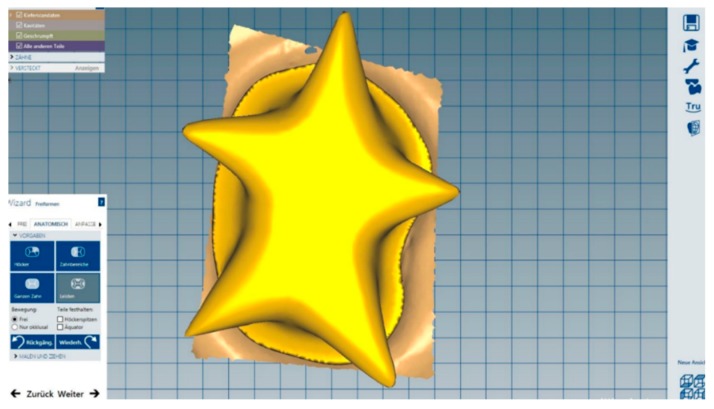
Star-shaped zirconia crown designing using Ceramill Mind software (Amann Girrbach).

Subsequently, a star-shaped crown was individually designed for each dentin abutment (Figure 6).

The crowns were milled (DD cubeX² 98, Dental Direkt, Spenge, Germany, Lot.No. 8041738003) with a 5-axis milling unit (Ceramill Motion 2, Amann Girrbach, Koblach, Austria). The zirconia crowns were sintered according to the manufacturer’s instructions (Nabertherm LHT 02/16, Nabertherm, Lilienthal, Germany) and air-abraded for 10 s with 50 µm aluminum oxide particles (pressure: 0.05 MPa; distance: 10 mm, and angle: 45°; basic Quattro IS, Renfert, Hilzingen, Germany). The crowns were cleaned for 4 min in an ultrasonic bath with distilled water (T-14 Transistor/Ultrasonic, L&R Manufacturing, Kearny, NJ, USA) and bonded with Scotchbond Universal (3M, Seefeld, Germany, Lot.No. 171004A) for 20 s and air-dried with a gentle air stream. Prior to bonding, all dentin abutments were refreshed using a fine grit round-end straight diamond bur (GEBR. BRASSELER, Lemgo, Germany, Lot.No. 126832) and randomly subdivided into 5 groups (Table 1; n = 12).

The application time was set at 3 min for all HA as recommended by the manufacturer. An air-water spray for exactly 30 s at a distance of 3 cm was used to rinse off the HA, subsequently the tooth was air-dried.

Retraction agents were classified by Nowakowska according to their pharmacological action into two major classes: Class 1 being adrenergics (vasoconstrictors) with α- and β-adrenegics or α-adrenegics only; and class 2 being astringents with chlorides or sulfates (Table 2) [26].

The predominance of astringents nowadays was demonstrated by several authors showing that aluminum chloride and ferric sulfate are the most commonly used HA [27,28]. The mechanism of action of AlCl_3_ to stop bleeding is astringent by precipitation of proteins. The acidic property of aluminum chloride causes a reaction with blood proteins, which in turn creates a barrier by coagulated proteins and thereby prevents the outflow of blood from vessels [29].

**Figure 7:**
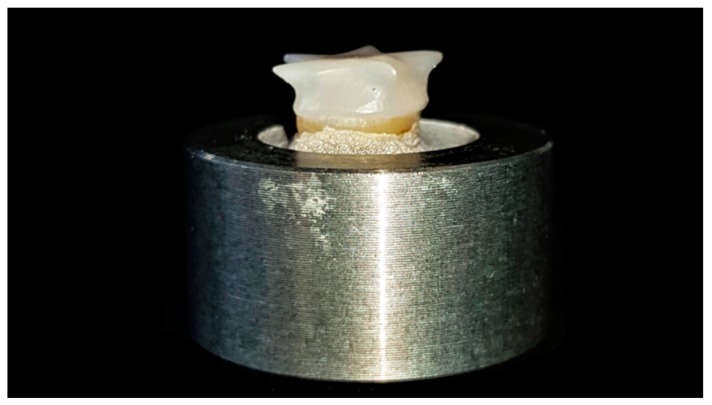
Adhesively luted zirconia crown.

Scotchbond Universal was applied for all groups as specified by the manufacturer: Rubbing onto the dentin for 20 s, air-drying for 5 s and light curing for 10 s using a light-curing unit (Bluephase, Ivoclar Vivadent, Schaan, Liechtenstein) with an output of 1200 mW/cm^2^. The dual-curing resin cement (RelyX Ultimate, 3M, Seefeld, Germany, Lot.No. 3382629) was applied to the crown, which was placed with finger pressure on the dentin abutment and light polymerized for 20 s from each side (occlusal, mesial, distal, buccal, oral; Bluephase; Ivoclar Vivadent, Schaan, Liechtenstein) (Figure 7).

**Figure 8:**
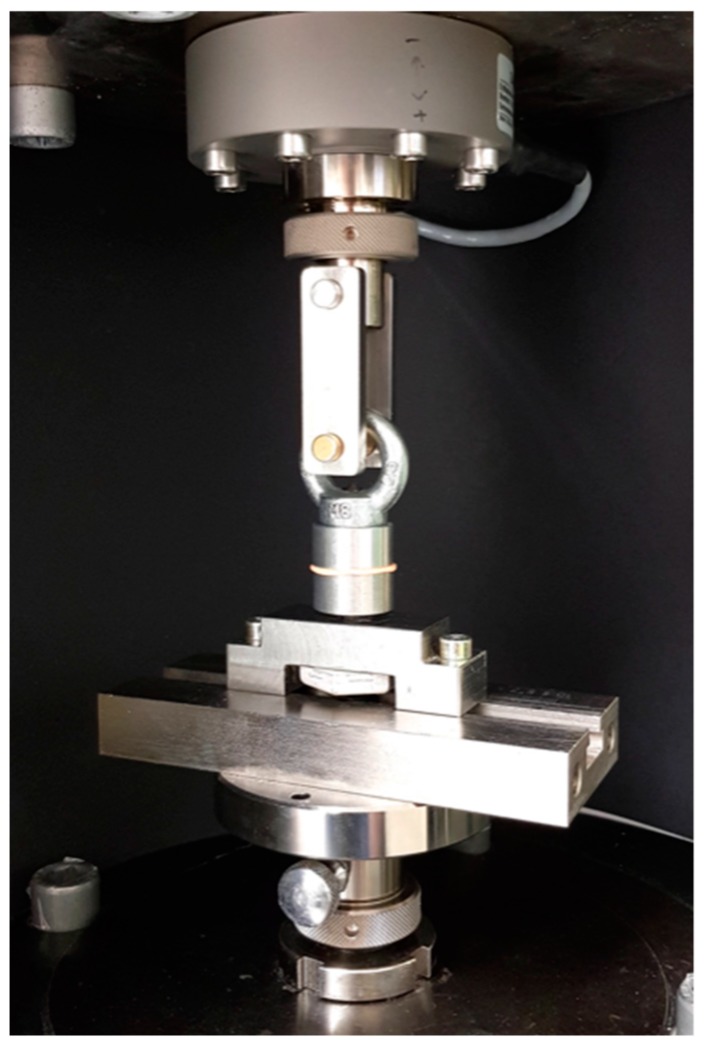
Testing specimens fixed into universal testing machine Zwick 1445 for pull out tests.

After cementation, specimens were placed in distilled water in an incubator (Hera Cell 150, Heraeus Kulzer, Hanau, Germany) at 37 °C for 24 h and were then mounted in a mastication simulator (CS-4, SD Mechatronik, Feldkirchen-Westerham, Germany) for 600,000 cycles with simultaneous thermocycling between 5° and 55 °C with a dwell time of 60 s. Ahead of the RS test, counterparts in form of the same aluminum bodies had to be placed to enable the fixation in the testing machine. A soft body polyvinyl siloxane (PVS) impression material (Flexitime Fast&Scan Light Flow, Kulzer, Hanau, Germany, Lot.No. R010022) was used as separating medium. Resin (ScandiQuick, SCAN-DIA, Hagen, Germany) was poured through the threaded opening of the counterpart embedding the crown. The specimens were fixed in the universal testing machine (1445 Zwick/Roell, Zwick, Ulm, Germany) and pulled with a crosshead speed of 5 mm/min until bond failure occurred (Figure 8).

RS was calculated with the following formula: retention strength = retention load/bonding surface (MPa = N/mm^2^).

The fracture types were determined with 10× magnification and 5 different fracture types were specified (Table 3).

The assumption of normality was tested using Kolmogorov–Smirnov test. One-way ANOVA followed by the post-hoc Scheffé test were used for comparison of the RS between the groups. The failure types were analyzed using Pearson’s chi-quadrat test and Ciba-Geigy table. The results of statistical analysis with p < 0.05 were interpreted as statistically significant. Data were analyzed with the computer software SPSS version 23.0 (IBM, Armonk, NY, USA).

## 3. Results

Two specimens showing fracture lines through the tooth roots (fracture type cohesive 1) were excluded from statistical analysis. The authors assume that in these specimens, the retention grooves were too extensive, weakening the root too much to withstand high pulling forces required for debonding of the zirconia crown. By this exclusion it was ensured that only forces acting on the bonding interface were evaluated.

**Figure 9:**
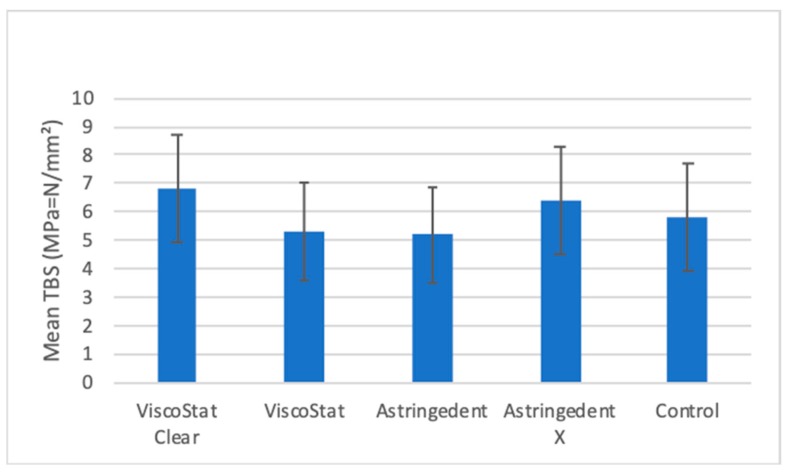
Mean RS values and their respective standard deviations for all groups.

No significant differences in RS between the tested groups were found (p = 0.205, Figure 9).

Table 4 demonstrates the relative frequencies of fracture types in all groups tested. Overall, adhesive 2 fractures seen in 36% of all specimens and cohesive 2 fracture in 34%, were the most common fracture types. Mixed fractures were seen in 17% of specimens and adhesive 1 fractures in 12%. In general, the control group showed the least number of cohesive failures when compared to groups contaminated with HA.

With ASX, cohesive type 2 fracture was seen in 50% (6) of specimens, while in control group it was only present in 16% (2). Vice versa, in control group mixed fracture was seen in 33% of specimens (4), while in ASX no single mixed fracture occurred.

VSC showed cohesive type 2 fracture in 41% (5) of specimens, but adhesive type 1 fracture only in 8% (1). VS showed adhesive type 2 fractures in 41% (5), but mixed fractures only in 8% (1) of specimens. AS showed cohesive type 2 fracture in 33% (4), but adhesive type 1 fracture in none of the specimens. Additionally, AS showed adhesive type 2 fracture in 50% (6) of specimens, but mixed fractures only in 16% (2). ASX showed cohesive type 2 fracture in 50% (6), but none mixed fractures.

## 4. Discussion

The null hypothesis was confirmed, because the comparison of RS did not show significant differences between the contaminated test groups and the control group, indicating that HA do not influence the RS of the tested adhesive luting procedure (Figure 9).

These results are only partly in concordance with previous studies concerning the RS of especially SEA in connection with HA. Most studies stated that HA negatively influence the RS of SEA. By comparing further studies, the main reason why it was not yet possible to draw conclusions was the lack of standardization regarding their methodology. The contamination technique varied from dropping the HA to application by a microbrush to even soaking the specimen into the HA with contamination times ranging from 10 s up to 48 h. In some studies, additional contamination by blood, saliva or a combination of both was applied prior to HA application. In addition, the used amount of HA may have an influence and has to be taken into account. Cleaning protocols to remove the agent prior to bonding varied from no cleaning at all to water rinses between 20 s and 5 min, to different cleaning methods using phosphoric acid, EDTA or aluminous oxide abrasion. The specimens themselves varied in different studies regarding their preparation (mostly prepared as dentin disks with composite resin cylinders adhesively luted to them), used dentin substrate (human primary/human permanent/bovine) and storing conditions. In almost none of the experiments an artificial aging process was implemented. The combination and interaction of different materials (adhesives, cements, HA) may also play a crucial role on the experiment´s outcome. All of these parameters of the methodology differing from the current study may have had an influence on the results. None of the previous studies resembled a situation close to a real clinic situation as seen in one- and also in two-appointment prosthetic procedures. Not only during one-appointment procedures, in which the final fixed dental prosthesis (FDP) is provided to the patient on the same day as the preparation of the tooth, but also during two-appointment procedures, in which a provisional FDP is provided until the final FDP is fabricated, the HA may have an influence on the luting procedure. Even in situations with a good provisional in combination with good oral hygiene, gingival hemorrhages may result from the removal of the provisional or the subsequent cleaning of the abutment. Especially in subgingival situations, the use of HA to assure a dry and clean working field previous to the final luting procedure could be necessary.

Therefore, by adjusting the parameters which possibly may have an impact on the outcome of the experiment leading to clinically better relevant data, the authors of the current study tried to simulate an as clinical as a possible situation. All specimens were prepared from human molar teeth according to the preparation standards of LMU Munich resembling abutment teeth as they could be found intraoral after crown preparation. Before the luting procedure, the dentinal surface of the teeth was refreshed using a diamond bur to ensure bonding conditions as in a freshly prepared tooth. The HA was applied as recommended by the manufacturer. A cleaning protocol of 30 s air/water spray was chosen as it is easily feasible and in the authors’ opinion the most commonly practiced cleaning method among dentists. Furthermore, SEA system and resin cement were chosen from the same manufacturer being recommended for the system usage, proven by a study in 2015 [30]. Therefore, the differences seen in material selection, specimen preparation, contamination, and cleaning, may have led to the outcome, that the tested HA did not influence the RS.

Different authors demonstrated changes in the dentinal smear layer and on the dentin surface after the HA application. One study showed that the dentinal smear layer appears to be replaced by a granular precipitate following the contamination with two different AlCl_3_-based HA (ViscoStat Clear; Hemodent). The authors assumed that the acidity of the HA (pH values of 0.7–3.0) [7,8,31] caused the dentin hydroxyapatite crystals to be solubilized, which bound with ionized calcium, resulting in an amorphous layer of calcium phosphates along with insoluble aluminum phosphates. In the same study the contamination with a Fe_2_(SO_4_)_3_-based HA (Astringedent) led to a dentin surface that appeared to be tufted with crystalline growths [11]. Further studies showed an increase of the aluminum content in dentin after the application of AlCl_3_-based HA. SEA were demonstrated to be not able to sufficiently reduce aluminum concentration in dentin. Calcium in hydroxyapatite may be replaced by aluminum resulting in the formation of Al(OH)_2_H_2_PO_4_, a hydroxyapatite form which is more resistant to acid etching. The deposition of unbound aluminum may result in a layer of residues and occlude dentinal tubules [9,10,12].

In addition, the application of HA may also result in the partial or complete removal of the dentinal smear layer [7,8,9,10]. Nevertheless, this demineralization effect exhibited by HA did not lead to a further enhancement of demineralization by SEA, instead the etching effect was reduced when compared to the control group, which may be explained by the incorporation of aluminum into the hydroxyapatite [9].

These authors therefore assumed that the relatively low acidity of SEA may neither be aggressive enough to remove all residual particles of HA from dentin nor strong enough to sufficiently etch a more acid-resistant dentin surface. In the current study none of these results and the resulting assumptions were proven right or wrong, but it is possible to assume that the combination of materials and/or the cleansing protocol used were able to overcome these described adverse effects of HA. Another factor which may influence the RS of adhesives to dentin, is the fact that dentin varies from tooth to tooth. The dentin tubule diameter changes during age and varies in size from the surface towards the pulp chamber [32]. The active and additional ingredients of the tested HA itself also may have affected the results of the experiment.

Only three previous studies concerning SEA tested the same HA as used in the current study. In 2011 one study showed a reduction in RS of a self-etching resin cement (RelyX Unicem, 3M) in combination with ViscoStat Clear. No adhesive system was used, specimens were contaminated by blood prior to a 5 min HA-application and the cleaning of 20 s air/water spray was shorter than in the current study [14]. Due to such differences it’s difficult to say what in particular led to the different outcomes. In another study two different SEA systems in combination with 1 min ViscoStat application followed by a 1 min air/water spray rinse were tested. The RS of one adhesive (AdheSE, Ivoclar Vivadent) was significantly reduced when compared to the control group. In contrast, with the other tested adhesive (AdheSE One F, Ivoclar Vivadent) no reduction in RS was demonstrated [16]. ViscoStat Clear, Astringedent and Astringedent X were further tested in combination with the adhesive Clearfil SE Bond and the resin cement Clearfil Photo Posterior (both Kuraray Noritake). A mixture of blood and saliva was applied to the dentin prior to HA application for 1 min followed by a 1 min water rinse. None of the agents had a detrimental effect on the RS [18]. These results enforce the assumption that the combination and interaction of specific materials may overcome or significantly reduce adverse effects of HA.

Unfortunately, the influence of HA on the adhesive Scotchbond Universal was not investigated in any other study, neither in combination with RelyX Ultimate nor any other resin cement. The assumption that Scotchbond Universal alone or in combination with RelyX Ultimate is therefore more resistant to changes caused by HA to dentin, when compared to other adhesives, could be made.

Failure types were mostly adhesive, seen in 48% (adhesive 1 + 2). Additionally, 17% of specimens showed a mixed bonding failure and 34% a cohesive bonding failure. It is common knowledge that cohesive failure types indicate high RS, while adhesive bonding failures indicate lower RS [33,34]. As the failure types distribution does not clearly point towards adhesive nor towards cohesive failure, it may be suggested that the RS between tooth and crown does not show differences regarding RS of the dentin substrate, indicating that HA contamination did not decrease the RS of the tested adhesive. Higher numbers of cohesive type 2 fracture in ASX when compared to the control group was observed. Within VSC and AS, significant higher numbers of cohesive type 2 fracture compared to adhesive type 1 and within ASX compared to mixed failure were found. Also, the control group shows the least total number of cohesive type 2 fractures when compared to contaminated groups. The authors therefore assume that the tested HA, especially Astringedent X, cause the dentin to become more brittle eventually resulting in more cohesive rather than adhesive failures. The actual etiology of this dentin weakening effect of tested hemostatic agents should be subjected to further studies. A limitation of this study was, that no morphological investigations at the dentine level were conducted, showing possible differences in the dentine substrate of different abutment teeth. The authors justify this lack of morphological investigation by pointing to the fact that the parameter of different dentine substrate morphology differs in every clinical situation and is not possible to influence by the practitioner.

## 5. Conclusions

The purpose of this study was to investigate, if hemostatic agents, which are inevitable for soft tissue management and ensure a clean and dry working field during prosthodontic procedures, have any detrimental effect on the adhesive bond between zirconia crowns and teeth.

Based on the findings of this in vitro study, three conclusions could be drawn. The first states that RS values did not show significant differences between the testing groups, indicating that contamination with HA does not have a negative influence on the performed adhesive luting procedure. The second conclusion states that cleaning the tooth for 30 s with an air/water spray seems to sufficiently remove HA, thereby preventing a detrimental effect on the bond strength between tooth and fixed dental prosthesis. The last conclusion is that the tested HA seem to have a weakening effect on dentin, making it prone to cohesive fracture. Nevertheless, this last point is just an assumption and needs to undergo further investigation in future studies involving microscopic examinations of the dentinal surface, as such weaknesses of the dentin might also be attributed to other factors such as the storage medium.

## Figures and Tables

**Table 1:** Overview of test groups with respective hemostatic agents.

Group	Active Ingredient	Product Name	Lot.No.
VSC	25% AlCl_3_	ViscoStat Clear *	BF61D
VS	20% Fe_2_(SO_4_)_3_	ViscoStat *	BF5X2
AS	15.5% Fe_2_(SO_4_)_3_	Astringedent *	BF3YR
ASX	12.7% iron ion solution containing ferric subsulfate and Fe_2_(SO_4_)_3_	Astringedent X *	BF1TC
control	-	-	-

* Manufacturer: Ultradent Products (Cologne, Germany).

**Table 2:** Overview of hemostatic agents (translated acc. Nowakowska [26]).

Class 1: Vasoconstrictors (Adrenergics)		Class 2: Astringents		
(α + β)-Adrenergics	α-Adrenergics	Aluminum-Chloride	Sulfur	
			Aluminum	Iron
0.1/1.0/8.0% adrenalin (e.pinephrine)	0.05% tetrahydrozoline HCL (e.g., Visine)	10% AlCl_3_ (e.g., Gingiva Liquid)	20% Al_2_(SO_4_)_3_ (e.g., Rastringent)	15.5% Fe_2_(SO_4_)_3_ (e.g., Astringedent)
-	0.05% oxymetazoline HCL (e.g., Afrin)	20% AlCl_3_ (e.g., Orbat, Alustin)	25% Al_2_(SO_4_)_3_ (e.g., Orbat Sensitive)	20% Fe_2_(SO_4_)_3_ (e.g., ViscoStat)
-	0.25% phenylephrine HCL (e.g., Neosynephrine)	25% AlCl_3_ (e.g., Racestyptine solution, ViscoStat Clear)	-	12.7% Fe_2_(SO_4_)_3_ + Ferric Subsulfate (e.g., Astringedent X)

**Table 3:** All classified fracture types.

Fracture Type	Description
Cohesive 1	Fracture line through tooth root (excluded from statistical analysis)
Cohesive 2	Fracture line through tooth crown
Adhesive 1	Cement adhering to tooth crown only
Adhesive 2	Cement adhering partly to tooth, partly to zirconia crown
Mixed	Mix of adhesive and cohesive failure

**Table 4:** Cross table showing fracture type distribution analyzed by Ciba-Geigy chart.

Group	Fracture Type				
	Cohesive 1 (Tooth Root) * (Absolute Number) (%) (95% Confidence Interval)	Cohesive 2 (Tooth Crown) (Absolute Number) (%) (95% Confidence Interval)	Adhesive 1 (Tooth Crown) (Absolute Number) (%) (95% Confidence Interval)	Adhesive 2 (Tooth/ZrO_2_) (Absolute Number) (%) (95% Confidence Interval)	Mixed (adh./coh.) (Absolute Number) (%) (95% Confidence Interval)
**ViscoStat Clear (i.)**	0	5	1	3	3
	0%	41%	8%	25%	25%
	(0;27)	(14;73)	(0;39)	(4;58)	(4;58)
**ViscoStat (ii.)**	1	3	2	5	1
	8%	25%	16%	41%	8%
	(0;39)	(4;58)	(1;49)	(14;73)	(0;39)
**Astringedent (iii.)**	0	4	0	6	2
	0%	33%	0%	50%	16%
	(0;27)	(8;66)	(0;27)	(20;79)	(1;49)
**Astringedent X (iv.)**	0	6	3	3	0
	0%	50%	25%	25%	0%
	(0;27)	(20;79)	(4;58)	(4;58)	(0;27)
**Control (v.)**	1	2	1	4	4
	8%	16%	8%	33%	33%
	(0;39)	(1;49)	(0;39)	(8;66)	(8;66)

* Cohesive 1 fracture type was not included into the statistical analysis.
